# Evaluating the clinical relevance of the enterotypes in the Estonian microbiome cohort

**DOI:** 10.3389/fgene.2022.917926

**Published:** 2022-08-17

**Authors:** Oliver Aasmets, Kertu Liis Krigul, Elin Org

**Affiliations:** Institute of Genomics, Estonian Genome Centre, University of Tartu, Tartu, Estonia

**Keywords:** metagenomics, gut microbiome, enterotypes, complex diseases, disease prediction

## Abstract

Human gut microbiome is subject to high inter-individual and temporal variability, which complicates building microbiome-based applications, including applications that can be used to improve public health. Categorizing the microbiome profiles into a small number of distinct clusters, such as enterotyping, has been proposed as a solution that can ameliorate these shortcomings. However, the clinical relevance of the enterotypes is poorly characterized despite a few studies marking the potential for using the enterotypes for disease diagnostics and personalized nutrition. To gain a further understanding of the clinical relevance of the enterotypes, we used the Estonian microbiome cohort dataset (*n* = 2,506) supplemented with diagnoses and drug usage information from electronic health records to assess the possibility of using enterotypes for disease diagnostics, detecting disease subtypes, and evaluating the susceptibility for developing a condition. In addition to the previously established 3-cluster enterotype model, we propose a 5-cluster community type model based on our data, which further separates the samples with extremely high *Bacteroides* and *Prevotella* abundances. Collectively, our systematic analysis including 231 phenotypic factors, 62 prevalent diseases, and 33 incident diseases greatly expands the knowledge about the enterotype-specific characteristics; however, the evidence suggesting the practical use of enterotypes in clinical practice remains scarce.

## 1 Introduction

Large-scale human microbiome studies have shown how the gut microbiome reflects our lifestyle and health (Zhernakova et al., 2016; Jackson et al., 2018; Gacesa et al., 2020; Aasmets et al., 2022). The implications on health and disease have particularly fed the growing interest in the microbiome field with the possibility of using the microbiome profile as a novel tool for disease diagnostics and microbiome-informed personalized therapeutics in mind (di Pierro, 2021; Zeevi et al., 2015). Moreover, recent studies show the potential of using the microbiome as a prognostic marker for disease progression, leading to personalized risk estimation (Aasmets et al., 2021; Liu et al., 2022; Ruuskanen et al., 2022; H. Wang et al., 2022). Taken together, the microbiome carries relevant information about one’s health which can be exploited for the benefit of public well-being. Nevertheless, there is no denying that the human microbiome is an exceptionally complex system, which is highly individualized, constantly undergoing changes and its characterization is demanding (Bartolomaeus et al., 2021; Vandeputte et al., 2021; Olsson et al., 2022). These properties have become obstacles to identifying robust signals, whether it is the identification of disease-associated microbes or the generalization abilities of complex diagnostic models (Wirbel et al., 2019; Nearing et al., 2022). A simple characterization of the complex microbial landscape to a small number of distinct clusters has been proposed as a possible solution (Arumugam et al., 2011). Although there is no consensus about the number of such distinct classes and even the existence of such clusters is being debated, a three-cluster “enterotype” model is perhaps the most well-known and discussed simplification of the inter-individual variability of the gut microbiome (Costea et al., 2017). Since the possibility of clusters of the gut microbiome was first described, their connection to human health has been of great interest and the knowledge is continuously growing. Additional motivation for using enterotypes is their relative intra-individual and temporal stability, which is a desired property for potential applications (di Pierro, 2021; Vandeputte et al., 2021). Even so, the clinical relevance of the enterotypes or related clusters is largely unclear, and several directions have been highlighted that need further research. Enterotypes have so far been considered for disease diagnostics (Zeller et al., 2014), personalized nutrition (Christensen et al., 2018), and implications for weight loss (Song et al., 2020; Zou et al., 2020), but their usage for estimating the risk of developing a condition, identifying differences in disease aetiologies and their implications for drug metabolism is poorly characterized (Costea et al., 2017).

Here, we provide a thorough phenotypic characterization of the subjects according to their fecal enterotype using the Estonian microbiome cohort dataset including shotgun metagenomic sequencing data from 2,509 individuals. The Estonian microbiome cohort takes advantage of electronic health records (EHR), which allows the characterization of the health and drug usage of the individual in great detail (Aasmets et al., 2022). We evaluate the possibility of using enterotypes for disease diagnostics, detecting disease subtypes, and evaluating disease risk using the available follow-up health data from EHRs. In addition, we proposed a 5-cluster model based on our data, which further separates the samples with extremely high *Bacteroides* and *Prevotella* abundances.

## 2 Materials and methods

### 2.1 Estonian microbiome cohort

The volunteer-based Estonian microbiome cohort (EstMB) was established in 2017 with the aim of enriching the data of the Estonian Biobank (EstBB) with microbiome data (Leitsalu et al., 2015; Aasmets et al., 2022). Stool, oral, and blood samples were collected from 2,509 EstBB participants (1,764 females and 745 males), aged 23–89 years. The detailed information about the sample collection and available data are described in Aasmets et al., 2022. All participants included in the EstMB provided informed consent for the data and samples to be used for scientific purposes. This study was approved by the Research Ethics Committee of the University of Tartu (approval No. 266/T10) and by the Estonian Committee on Bioethics and Human Research (Estonian Ministry of Social Affairs; approval No. 1.1-12/17). All participants have joined the Estonian Biobank on a voluntary basis and have signed a broad consent form, which allows receiving participants’ personal and health data from national health registries and databases. The rights of gene donors are regulated by the Human Genes Research Act (HGRA) § 9–Voluntary nature of gene donation (https://www.riigiteataja.ee/en/eli/ee/531102013003/consolide/current).

For the current analysis, arbitrary selection of diseases (based on ICD10 categories) with at least 20 cases were chosen for downstream analysis, resulting in 62 prevalent diseases and 33 incident diseases (Supplementary Table S1A). Medications were grouped into categories based on Anatomical Therapeutic Chemical classification (ATC codes) at the highest ATC level (up to 7-digit code—ATC level 5). ATC categories with less than 20 cases were grouped into a higher level. ATC categories with less than 20 cases at any ATC level were removed from the analysis. In total, 122 medications or medication groups were analyzed, out of which 81 were classified at the ATC level 5 (7-digit code), 26 were classified at the ATC level 4 (5-digit code) and 56 were classified at the ATC level 3 (4-digit code) (Supplementary Table S1A). In addition to the electronic health records data, the patients reported their diseases, medications, medical procedures, and health behavior in lifestyle and microbiome study-specific questionnaires, which included questions about their diet (e.g., dietary frequency questionnaire), physical activity, medical data, living environment, delivery mode and stool characteristics (Bristol stool scale). The analyzed factors are listed in Supplementary Table S1A.

### 2.2 Microbiome sample collection and DNA extraction

The participants collected a fresh stool sample immediately after defecation with a sterile Pasteur pipette and placed it inside a polypropylene conical 15 ml tube. The participants delivered the sample to the study center where it was stored at -20°C until DNA extraction. Microbial DNA extraction was performed using QIAamp DNA Stool Mini Kit (Qiagen, Germany). For the extraction, around 200 mg of stool was used as a starting material following the DNA extraction kit manufacturer’s instructions. DNA was quantified from all samples using Qubit 2.0 Fluorometer with dsDNA Assay Kit (Thermo Fisher Scientific). NEBNext^®^ Ultra™ DNA Library Prep Kit for Illumina (NEB, United States) was used for generating sequencing libraries following the manufacturer’s recommendations. Briefly, 1 μg DNA per sample was used as input material. Index codes were added to attribute sequences to each sample. The DNA sample was fragmented by sonication to an average size of 350 bp, DNA fragments were end-polished, A-tailed, and ligated with the full-length adaptor for Illumina sequencing with further PCR amplification. Finally, PCR products were purified (AMPure XP system) and libraries were analyzed for size distribution by Agilent2100 Bioanalyzer and quantified using real-time PCR.

### 2.3 Metagenomics data analyses

The shotgun metagenomic paired-end sequencing was performed by Novogene Bioinformatics Technology Co., Ltd., using Illumina NovaSeq6000 platform resulting in 4.62 ± 0.44 Gb of data per sample (insert size 350 bp, read length 2 × 250 bp). A total of 2,509 samples were sequenced. First, the reads were trimmed for quality and adapter sequences. The host reads which aligned to the human genome were removed using SOAP2.21 (parameters: -s 135 -l 30 -v 7 -m 200 -x 400, Li et al., 2009). Quality-controlled data of each sample was then used for metagenomic assembly using SOAPdenovo (v. 2.04, parameters: -d 1 -M 3 -R -u -F, Luo et al., 2012). SOAP2.21 was then used to map clean data of each sample to the assembled scaftigs (i.e., continuous sequences within scaffolds). Unutilized paired-end reads of each sample were put together for mixed assembly. MetaGeneMark (v.3.38, http://exon.gatech.edu/meta_gmhmmp.cgi) was used to carry out gene prediction (gene length > 100 bp) based on the scaftigs (≥ 500 bp) which were assembled by single and mixed samples. CD-HIT (v.4.6) was used to dereplicate the predicted genes based on 95% identity and 90% coverage to generate gene catalogues (parameters: -c 0.95, -G 0, -aS 0.9, -g 1, -d 0) (W. Li & Godzik, 2006). The longest dereplicated gene was defined as the representative gene (i.e., unigene). SoapAligner (v.2.21, parameters: -m 200, -x 400, identity ≥ 95%) was then used to map clean data to gene catalogs and to calculate the quantity of the genes for each sample (Gu et al., 2013). The gene abundance was calculated based on the total number of mapped reads and normalized gene length. The taxonomic composition of metagenomes was identified by comparing marker gene homologs to an NR database (201810) of taxonomically informative gene families using DIAMOND (v0.9.9.110, Buchfink et al., 2014). The homologs were annotated based on the sequence or phylogenetic similarity to the database sequences.

### 2.4 Filtering and preprocessing microbiome data

For downstream analysis, we removed three samples with an exceptionally low number of reads resulting in 2,506 samples. In total, 17,158 species were identified. Species that were detected with > 10% prevalence at a relative abundance of 0.1% were used, resulting in 1,231 species. Next, the taxonomic table was aggregated to the genus level before community typing resulting in 226 genera.

### 2.5 Statistical analysis

All statistical analyses were carried out using the R (v. 4.1.1) software.

#### 2.5.1 Identifying enterotypes and community types

**FIGURE 1:**
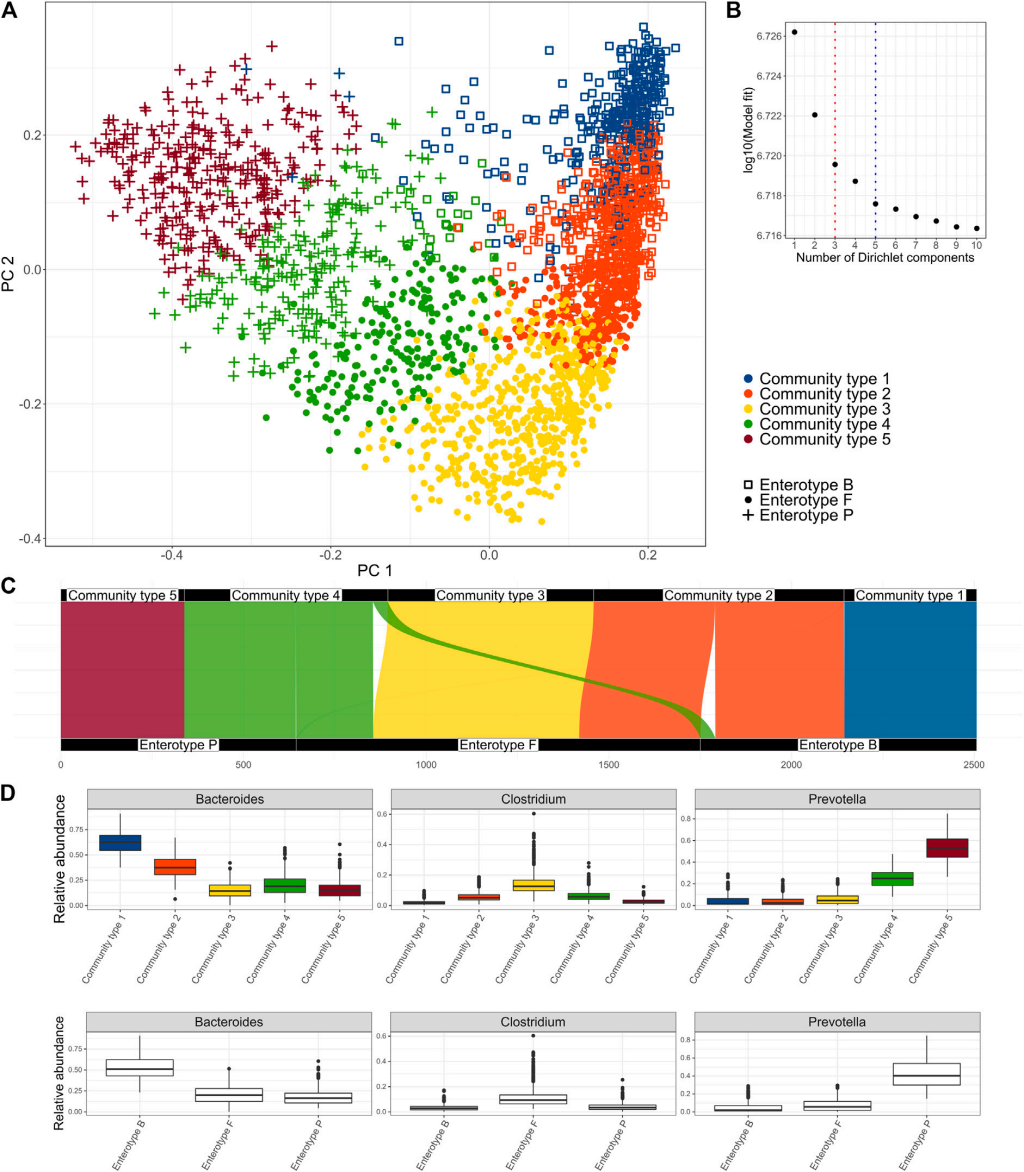
Clusters identified in the Estonian microbiome cohort metagenome data obtained by the Dirichlet Multinomial Mixture Model. **(A)** enterotypes and community types on the PCoA biplot of the species-level microbiome profile based on the Bray-Curtis dissimilarity, **(B)** Model fit by the number of clusters; 3 clusters represent the enterotype (ET) model and 5 clusters were selected as an optimal number (community type CT model), **(C)** Correspondence of the clusters obtained by the CT model with the clusters obtained by the ET model, **(D)** relative abundances of the driving genera by the community types and enterotypes.

The Dirichlet Multinomial Mixture model was applied to the genus level microbial community profiles using the *DirichletMultinomial* package (v1.34.0) (Holmes et al., 2012). Genus level taxonomy was used to allow compatibility with other studies and because it is hypothesized that at genus level the ecological niches are most clearly reflected (Dethlefsen et al., 2006). Up to ten clusters were considered and the number of clusters that best fit the data was determined using Laplace approximation. As the number of optimal community types selected by the model depends on the sample size, we considered optimal the number of clusters after which there was no significant improvement in the model fit (Figure 1B). For detecting enterotypes, three clusters were chosen based on the same methodology.

#### 2.5.2 Association analysis

Logistic and linear regression models were used to associate the binary and continuous factors with the community types using the *glm* and *lm* functions. In addition to the enterotypes and community types, the log-transformed *Prevotella*-*Bacteroides* ratio was used for comparison to represent the “gradient-model.” Categorical variables were associated with the cluster composition using the chi-squared test. We corrected for multiple testing using the Benjamini–Hochberg FDR correction. Additionally, multivariate logistic regression models adjusted for gender, age, body mass index (BMI), and stool consistency were used for assessing the clusters for diagnostic applications. Likelihood ratio tests were used to analyze whether including the cluster in the model improves the model fit. Furthermore, posthoc tests were carried out to test whether drug usage confounds the associations between the diseases and clusters. The disease-clusters association was considered confounded if at least one drug was found such that the cluster did not further improve the model fit in addition to the drug.

Associations between the incident diseases and clusters were analyzed using the Cox proportional hazard models using the *survival* package (v3.2.11) after adjusting for baseline age, BMI, gender, and stool consistency. For each diagnosis analyzed, the subjects with prevalent diseases were excluded from the analysis. The median follow-up time is 3.1 years. The proportional hazards (PH) assumption was tested using the *cox.zph* function (Supplementary Table S4A). For analyzing differences in the distributions of subdiagnosis, the chi-squared test was used.

## 3 Results

### 3.1 Community types in the Estonian microbiome cohort data

First, we aimed to identify clusters from the Estonian microbiome cohort gut metagenomics data (*N* = 2,506). We applied the Dirichlet Multinomial Mixture (DMM) model to the genus-level taxonomic profile, as DMM can help to infer the number of clusters in the data (Holmes et al., 2012). The selection of the number of clusters can be influenced by the sample size. Thus, we focused on the model that selected five clusters as there was no further significant improvement in the model fit (Figures 1A,B). We refer to the 5-clusters as community types (*CT*) model and indicate the clusters accordingly (*CT1*-*CT5*). In addition, we analyze the 3-cluster enterotype (*ET*) model, which has been most consistently reported to describe structures in the fecal microbiome. The driving genera of the *CT* model and the relative abundances of the most important genera *Bacteroides*, *Prevotella,* and *Clostridum* by the community types are shown in Figure 1D and Supplementary Figure S1. Comparing the clusters obtained by the two models shows that community types *CT1* and *CT5* belong almost exclusively to enterotypes defined by the dominance of *Bacteroides* (*ET B*) and *Prevotella* (*ET P*) respectively. Community types *CT2*, *CT3,* and *CT4* are divided between multiple enterotypes with the enterotype dominated by genera from *Firmicutes* (*ET F*) consisting of samples from all 3 community types (Figure 1C). Taken together, the community-type model further separates samples with an extremely high relative abundance of *Bacteroides* (CT1) and Prevotella (CT5). Differences in the microbiome characteristics (e.g., observed species and Shannon diversity) of the community types and enterotypes are consistent with the enterotype—community type transition (Figure 1C, Supplementary Figure S2). Consistent with previous results, *ET F* shows the highest taxonomic and functional diversity, but also the highest richness of antibiotic resistance genes (ARGs) while *ET B* shows the lowest taxonomic and functional richness (Supplementary Figure S2) (Costea et al., 2017). Shannon diversity index was similar between *ET B* and *ET P*. Being the most dominated by the *Bacteroides* and *Prevotella* (Figure 1D), community types *CT1* and *CT5* show significantly lower diversity compared to other community types. As previously reported, enterotype *ET B* and *ET P* are associated with looser stools and higher gut emptying frequency (Supplementary Table S1B, Supplementary Figure S3). On the other hand, *ET F* is associated with dry stools, less frequent gut emptying and more self-reported constipation (Supplementary Table S1B, Supplementary Figure S3). The gut emptying frequency and stool consistency for community types follow a concordant pattern regarding enterotype-community type transitions with *CT3* being associated with constipation and less frequent gut emptying, *CT1* and *CT5* having loose stools and higher frequency, and *CT2* and *CT4* falling consistently on the gradient.

### 3.2 Characterization of the phenotypic associations with the enterotypes and community types

**FIGURE 2:**
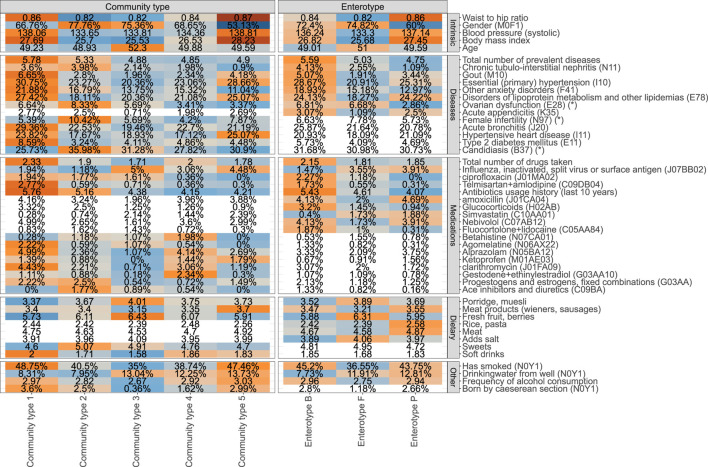
Phenotype associations with the enterotype (ET) model and community type (CT) model (unadjusted analysis). Coloured cells represent factors associated with CT and ET models respectively (FDR ≤0.1), and white cells indicate no statistically significant association (FDR > 0.1). Mean values or proportions (indicated by %) per cluster are shown. Blue colors indicate lower mean values or proportions for the cluster and orange color indicates higher values. Asterix (*) in the names of the factors indicate that a subpopulation consisting of women was used for calculating the displayed value.

Next, we analyzed the associations between 231 phenotypic factors, enterotypes and proposed community types (Supplementary Table S1A). These factors include 62 diseases, 125 medications, 3 clinical procedures, 20 dietary items, 5 intrinsic factors, and 16 other factors characterizing the lifestyle. We identified 36 factors associated with the enterotypes and 42 factors associated with community types after correcting for multiple testing (FDR≤0.1) and 53 and 60 factors respectively with nominal significance (*p*-value ≤0.05) (Figure 2, Supplementary Figure S3, Supplementary Table S1A). Out of the associations which were significant with FDR ≤0.1, 25 factors were associated with both enterotypes and community types, 11 associations were specific to enterotypes, and 17 associations were specific to community types.

The identified associations with enterotypes reveal a homogenous picture of one’s health (Figure 2). Overall, *ET B* is associated with deteriorated health represented by the highest average number of prevalent diseases and highest number of different medications used. The diseases showing the most significant enrichment in *ET B* include gout, primary hypertension, anxiety, and chronic tubulo-intestinal nephritis, but suggestive evidence (nominal *p*-value ≤ 0.05) shows enrichment in *ET B* similarly for several other diseases, most notably for major depressive disorder (Supplementary Tables S1A,B). Enterotype *ET P* on the other hand corresponds to best health in terms of the number of prevalent diseases. Subjects from *ET P* and *ET B* enterotypes show similar associations with physical characteristics and lifestyle parameters such as high blood pressure, BMI, waist-to-hip ratio (WHR), higher rate of smokers, and higher frequency of drinking alcohol when compared to the subjects from *ET F* (Figure 2, Supplementary Table S1B). Therefore, *ET F* seems to represent subjects with the healthiest lifestyle, which is further supported by the least number of medications used and a low prevalence of diseases such as gout and primary hypertension. There is also a significant association with gender and age with *ET P* showing a significantly lower proportion of women and the subjects from *ET F* are slightly older than the others. Importantly, the consumption of antibiotics and especially long-term usage of antibiotics characterized by the number of prescriptions bought in the last 10 years before sample collection is associated with the enterotype composition. The subjects from *ET B* have used significantly more antibiotics in the previous years when compared to the subjects from *ET F* and *ET P* (Figure 2, Supplementary Table S1B).

Although the 3-cluster model of enterotypes clearly corresponds to differences in health and lifestyle, the 5-cluster community type model leads to even more distinct phenotypic profiles. Notably, in addition to more factors being associated with the community types when compared to enterotypes, the 5-cluster *CT* model highlights a mixture of possible association patterns. Some associations are specific to certain community types, and some exhibit a gradient-like nature. For example, the prevalence of type 2 diabetes shows a clear enrichment that is specific to a community type *CT1*. Correspondingly, the community type model for type 2 diabetes shows the best fit in terms of the Akaike Information Criterion (AIC) when compared to the enterotype model and the gradient model (Supplementary Table S1A). Similarly, associations with hypertensive heart disease, asthma, and even female fertility show associations which are most concordant with the community types (Figure 2). Importantly, age, BMI, WHR, and blood pressure are also most concordant with the community type model. On the other hand, some factors associated with the microbiome in a gradient-like manner, corresponding to the *Bacteroides*-*Prevotella* ratio (Supplementary Figure S4). For example, the average number of courses of antibiotics taken over the previous 10 years increases from *CT3* to *CT1*, with no clear-cut community type-specific association as for type 2 diabetes, thus also best represented by the gradient model in terms of AIC. Similarly, multiple dietary factors and host-targeted medications were found to be associated with enterotypes and community types that also show signals specific to the clustering scheme used.

### 3.3 Assessing the clinical relevance of the enterotypes and community types

Next, we aimed to assess the potential clinical relevance of the enterotypes and community types, concentrating on the diseases. The vast number of phenotypic associations is a valid motivation for considering the diagnostic application. However, the common risk factors (BMI, age, gender, alcohol, smoking) for many complex diseases are also integral characteristics in distinguishing the clusters. Also, drug consumption can complicate the identification of disease-specific signals (Forslund et al., 2021). Therefore, we adjusted the models for prevalent diseases for age, BMI, gender, and stool consistency to understand, whether the associations as shown can be confounded by these covariates. After adjusting, only associations with gout, disorders of lipoprotein metabolism, essential hypertension, and chronic tubulo-interstitial nephritis for the enterotype-model and gout and anxiety disorders for the community type model remained statistically significant (Supplementary Table S1A). Next, we asked, whether these associations are confounded by drug usage. When further adjusted for drug usage, associations with anxiety disorder and tubulointerstitial nephritis were not detected (Supplementary Table S2).

We further asked the question of whether the clusters can identify differences in disease etiology. Instead of the 3-digit ICD10 codes, we focused on the disease subcodes and analyzed, whether there are differences in the distribution of the occurrences between the identified clusters (Supplementary Table S3A, Supplementary Figure S5). We found suggestive evidence for differences in the sub-diagnosis distributions for Gastritis and duodenitis (K29) with chronic superficial gastritis (K29.3) being more prevalent in *CT2* and for disorders of lipoprotein metabolism (E78) with mixed hyperlipidemia (E78.2) being more characteristic to *ET F* and pure hyperlipidemia to *ET P* and *ET B*. Finally, we analyzed the incident diseases with the aim assessing the enterotype-like approaches for assessing the susceptibility for developing a disease. We applied Cox proportional hazard models to the incident cases, adjusting for baseline age, BMI, gender, and stool consistency. After analyzing 33 incident diseases, we found only suggestive evidence for predicting migraine using the 5-cluster community type model (*p*-value: 0.0431) with CT3 showing decreased risk when compared to the other community types (Supplementary Tables S4A,B, Supplementary Figure S6)

## 4 Discussion

High inter-individual and temporal variability of the gut microbiome can undermine the development of microbiome-based applications in personalized medicine. Thus, collapsing the microbiome profiles into a small number of clusters has desirable properties for summarizing and communicating the role of the microbiome in human health. That is why the concept of enterotypes is still being actively discussed and researched, 10 years after its first mention (Costea et al., 2017; di Pierro, 2021). Nevertheless, the prospect of using enterotypes for disease diagnostics and disease risk estimation remains largely unknown. Here, using the comprehensive gut metagenome and health data from the Estonian microbiome cohort (EstMB), we characterized the phenotypic differences between the subjects from different enterotypes. Taking advantage of the electronic health records, in addition to identifying novel associations between enterotype composition and various lifestyle factors, we were able to show that the enterotypes can discriminate disease subtypes. Furthermore, our data suggest that a 5-cluster model can provide a more comprehensive look on the lifestyle and health by identifying subjects with elevated risks for developing incident diseases while retaining simplicity and explainability. The microbiome composition undergoes the most rapid developments in early childhood and constantly changes throughout the adulthood with diet and lifestyle being one of the most influential factors for the underlying changes (Gilbert et al., 2018; Dinsmoor et al., 2021). Similar dynamics and influential factors go hand in hand with the enterotype composition (Dinsmoor et al., 2021). Therefore, it is not surprising that the dietary items such as the consumption of porridge, fresh fruit, meat, and indications of lifestyle such as drinking water origin, alcohol consumption, and smoking were associated with the enterotype composition in the Estonian microbiome cohort. The dominance of the *ET B* enterotype in the urban region and the association of *ET B* with a diet rich in animal proteins and saturated fats is well known (de Filippo et al., 2010; Ley, 2016). Yet, our results regarding the diet are conflicting. It is possible that the food-frequency questionnaire doesn’t allow to characterize the diet in necessary detail. Also, enterotypes have been associated with body composition. For example, both *ET P* and *ET B* enterotypes have been associated with a higher waist circumference and *ET B* with a higher BMI (Breuninger et al., 2021). Our results confirm these observations, but additionally, show that the *ET P* is associated with higher BMI when compared to *ET F*. Interestingly, subjects belonging to *ET F* tend to be slightly older than the subjects from *ET B* and *ET P* and there was a significantly lower proportion of women in *ET P*. Furthermore, we show that the *CT* model further emphasizes the differences in body composition and lifestyle. Taken together, there are remarkable differences in diet and lifestyle between the subjects from different enterotypes and community types, that can have an impact on the enterotype-focused applications and must be accounted for.

Besides the lifestyle, the associations between enterotypes and complex diseases have gained special interest due to the potential for direct diagnostic application. Previously, enterotypes have been associated with numerous diseases such as dementia (Saji et al., 2019) and colorectal cancer (Zeller et al., 2014). It is noteworthy that in these studies the anthropometric measurements and lifestyle factors, which are common confounders in microbiome studies (Vujkovic-Cvijin et al., 2020), are often not adjusted for. Correspondingly, after adjusting for gender, BMI, age, and stool consistency, we were able to confirm associations only with gout, disorders of lipoprotein metabolism, essential hypertension, anxiety disorder, and chronic tubule-interstitial nephritis. Furthermore, recent studies show that the drugs used to induce significant changes in the gut microbiome composition and complicate the identification of disease-specific signals (Forslund et al., 2021). After further adjusting for drug usage, the associations with chronic tubule-interstitial nephritis and anxiety disorders disappeared, but other associations remained significant indicating a stronger disease-specific association with the clusters. Thus, our data confirm that the enterotypes can have the property of distinguishing the healthy from the diseased. Moreover, our results indicate that the enterotypes and community types can help to distinguish differences in disease aetiologies, which is an important implication for future studies. Nevertheless, the lifestyle and anthropometric differences between the enterotypes need to be adjusted for to assess whether the enterotypes are a viable option for diagnostics. Also, given the same enterotypes and community types are linked to different diseases, the enterotyping alone might not be sufficient for diagnostic purposes (Costea et al., 2017).

In addition to considering enterotypes for disease diagnostics, the electronic health records allowed us to assess the susceptibility of developing a disease depending on the clusters. The research on enterotypes and differing health risks is rather scarce. Previously, *ET B* has been shown to be a risk factor for type 2 diabetes due to decreased insulin sensitivity (J. Wang et al., 2020). Also, *ET P* has been shown to have a lower risk for developing Parkinson’s disease (Heinzel et al., 2021). However, our analysis didn’t show any statistically significant results with any of the analyzed conditions after the FDR correction. Nominally significant differences in disease risk were identified only for migraine in the case of the 5-cluster model with *CT3* showing the lowest risk for migraine. Therefore, although a simple clustering scheme is attractive and easily communicable, it might not be fit for estimating the disease risks. The stability of the enterotype composition has been considered its strength for risk assessment, but recent research suggests that the enterotype composition might be less stable than previously thought, which can explain our results (Olsson et al., 2022).

The concept of enterotypes or distinct clusters in the gut microbiome and the number of the clusters has been argued for and against without a clear consensus (Costea et al., 2017). Besides the original 3-cluster approach, several other clustering strategies and methodologies have been used, which have identified a varying number of clusters (Claesson et al., 2012; Zhou et al., 2014). Different clusters on the other hand can highlight distinct aspects of health. Therefore, criticism has accompanied the clustering approach and even a gradient model has been proposed instead (Koren et al., 2013). However, different aims need to be kept in mind when clustering microbiome data is carried out. First, whether there are distinct clusters in the microbiome can be a viable question. Second, we can ask whether the clusters we identify can be beneficial for our cause? We argue that if the second cause is kept in mind, then the replicability of the clustering is not the primary aim, and we encourage carrying out a *de novo* clustering on the dataset. It is possible that the clusters and therefore their practical properties are specific to the study population. We identified a 5-cluster model that provided a more distinctive characterization of the phenotypic profile when compared to the enterotype model. Even though we identify only weak signals for estimating disease risks and differences in disease aetiologies, the 5-cluster model implicated a more comprehensive approach for practical purposes when compared to the enterotype model. Thus, depending on the aim of the application, the *CT* model, which further emphasizes the “extremes” of the *Bacteroides*-*Prevotella* gradient, can be considered as an alternative to the enterotype model. Taken together, the 5-cluster model can be more beneficial for disease classification, disease risk estimation, and provide additional value for evaluating the overall health while maintaining simplicity.

Some limitations need to be acknowledged. Although the data in the electronic health records are comprehensive and of high quality, the subphenotypes are not that well-characterized with a large proportion of the diseases classified to a subcode indicating an unspecified condition. However, we were still able to identify differences in the cluster composition for some subphenotypes, which highlights the necessity for further research. Electronic health records allow to track the participant’s health over time and analyze incident diseases. However, the median follow-up time for the participants of our study is currently around 3 years, which allowed us to study the short-term risk of disease occurrences. Future studies can take advantage of the increased follow-up time and the perspective of using enterotypes for disease risk assessment can be revisited. Also, the Estonian microbiome cohort currently includes only one timepoint, therefore the potential enterotype or community type shifts cannot be studied. Undoubtedly, longitudinal data can improve the understanding of the community structures and evaluating the stability of the proposed 5-cluster model is necessary.

Taken together, clustering the microbiome data possesses admirable properties and such simplification would be highly valuable for communicating the microbiome science and for giving microbiome-informed personalized health information. Nevertheless, the evidence for using the enterotype-like clusters for clinical applications remains fragile.

## Data Availability

The metagenomic data generated in this study have been deposited in the European Genome-Phenome Archive database (https://www.ebi.ac.uk/ega/) under accession code EGAS00001008448. The phenotype data contain sensitive information from healthcare registers, and they are available under restricted access through the Estonian biobank upon submission of a research plan and signing of a data transfer agreement. All data access to the Estonian Biobank must follow the informed consent regulations of the Estonian Committee on Bioethics and Human Research, which are clearly described in the Data Access section at https://genomics.ut.ee/en/content/estonian-biobank. A preliminary request for raw metagenome and phenotype data must first be submitted via the email address releases@ut.ee. Used databases are NCBI nonredundant (NCBI nr) database 201810 (https://www.ncbi.nlm.nih.gov/blast/db/) and KEGG (https://www.kegg.jp/).
