# Supplementary material for: Evaluating the clinical relevance of the enterotypes in the Estonian microbiome cohort
Source: Front Genet. 2022 Aug 17;13:917926. doi: 10.3389/fgene.2022.917926 (PMC9428584; doi:10.3389/fgene.2022.917926)
Supplement: Supplementary file 1 [file DataSheet1.DOCX]

Supplementary Material


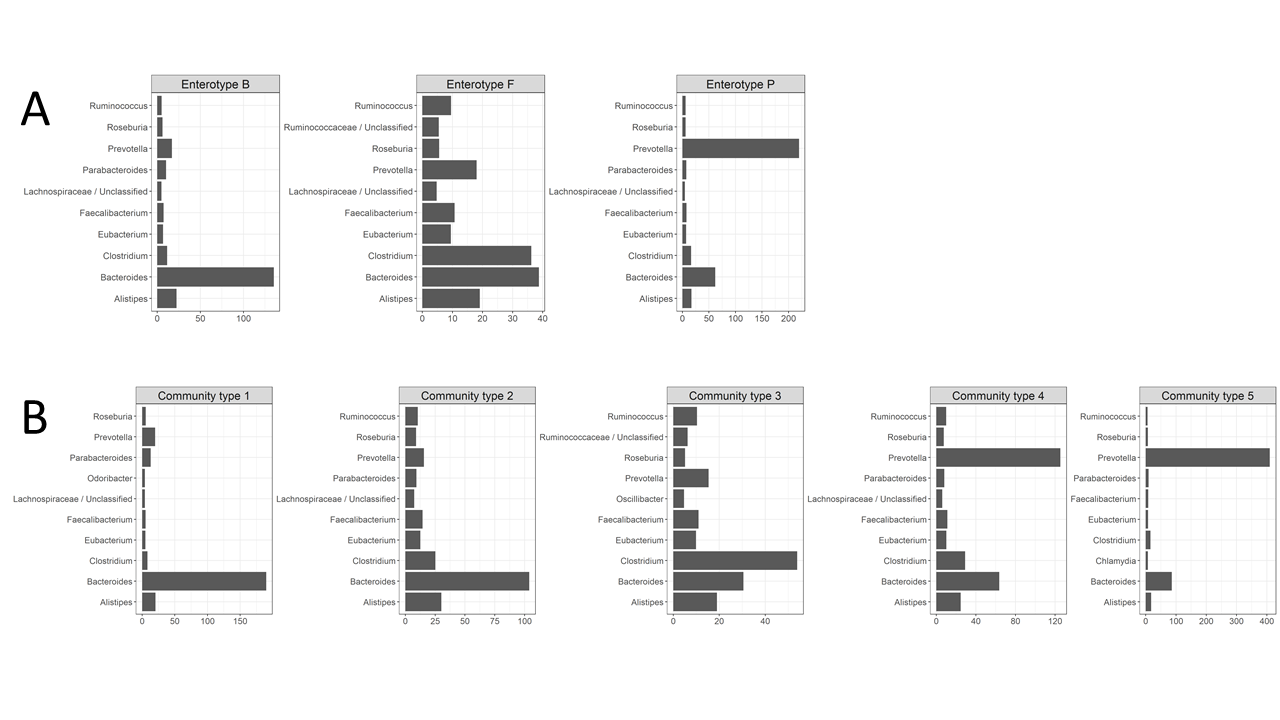


**Supplementary Figure 1.** Genera contributing most to the Dirichlet components for the enterotype model (A) and for the community type model (B).


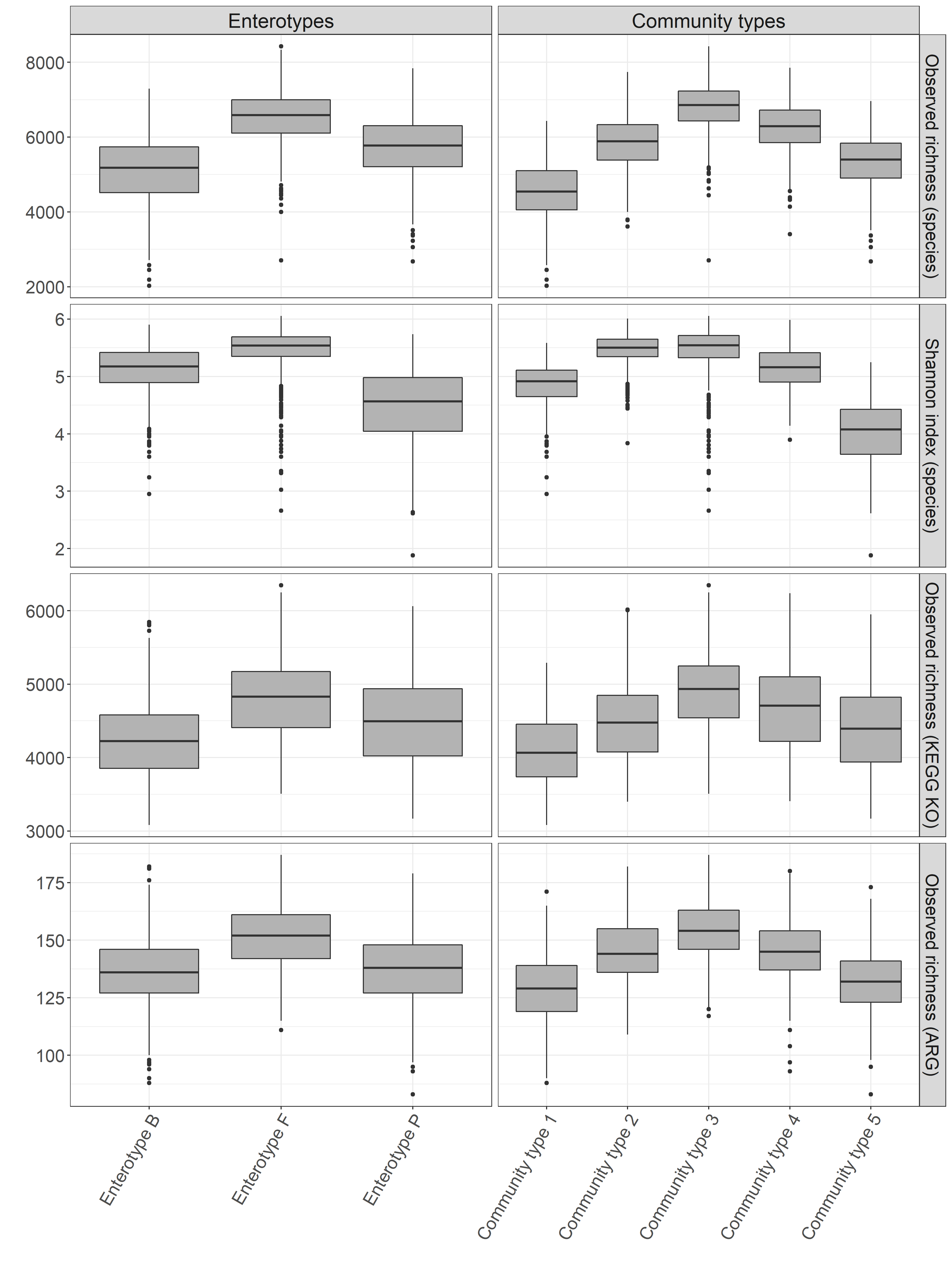


**Supplementary Figure 2.** Functional characteristics of the obtained enterotypes and community types.


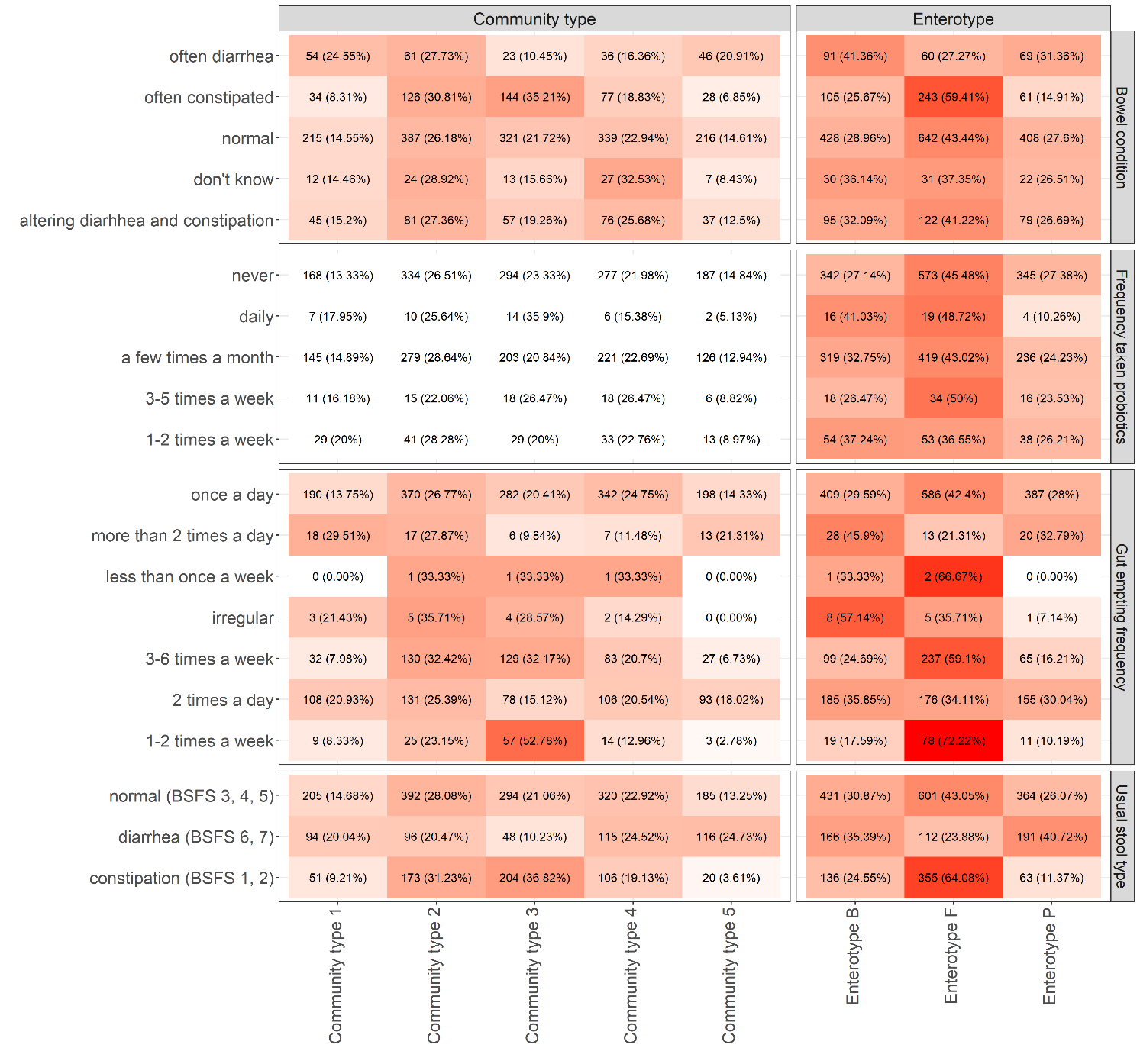


**Supplementary Figure 3.** Distributions of the categorical variables that were identified to be significantly associated with the enterotype (ET) or community type (CT) model. Cells are colored for factors found to be associated with *CT* and *ET* models respectively (FDR <= 0.1). The intensity of the color represents the row-normalized proportion of the answer. Frequency taken probiotics was not found to be associated with the community types (FDR > 0.1).


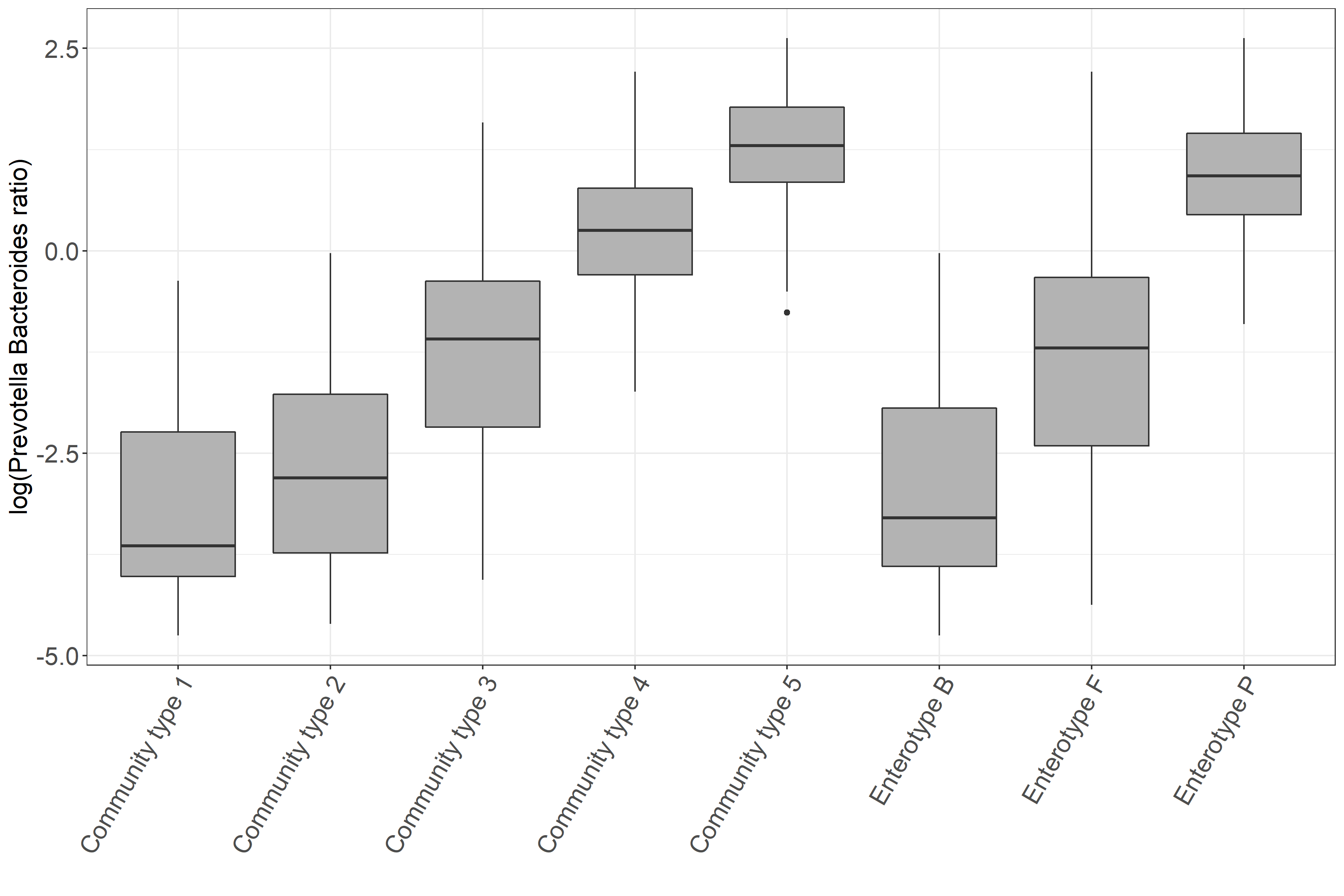


**Supplementary Figure 4.** *Prevotella*-*Bacteroides* ratio by enterotypes and community types.


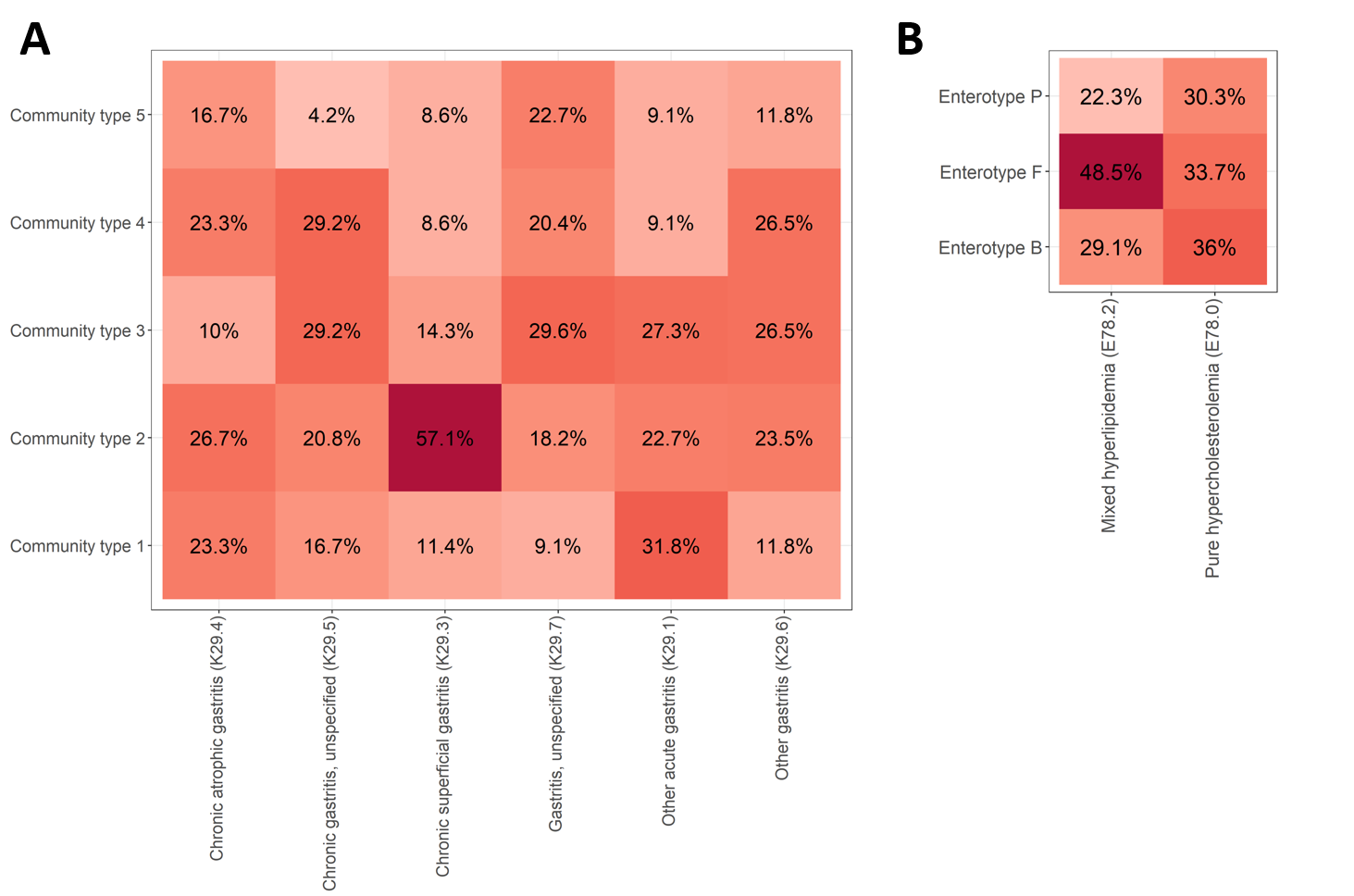


**Supplementary Figure 5.** Distribution of disease subtypes for **A -** Gastritis and duodenditis (K29) and **B -** Disorders of lipoprotein metabolism and other lipidemias (E78)


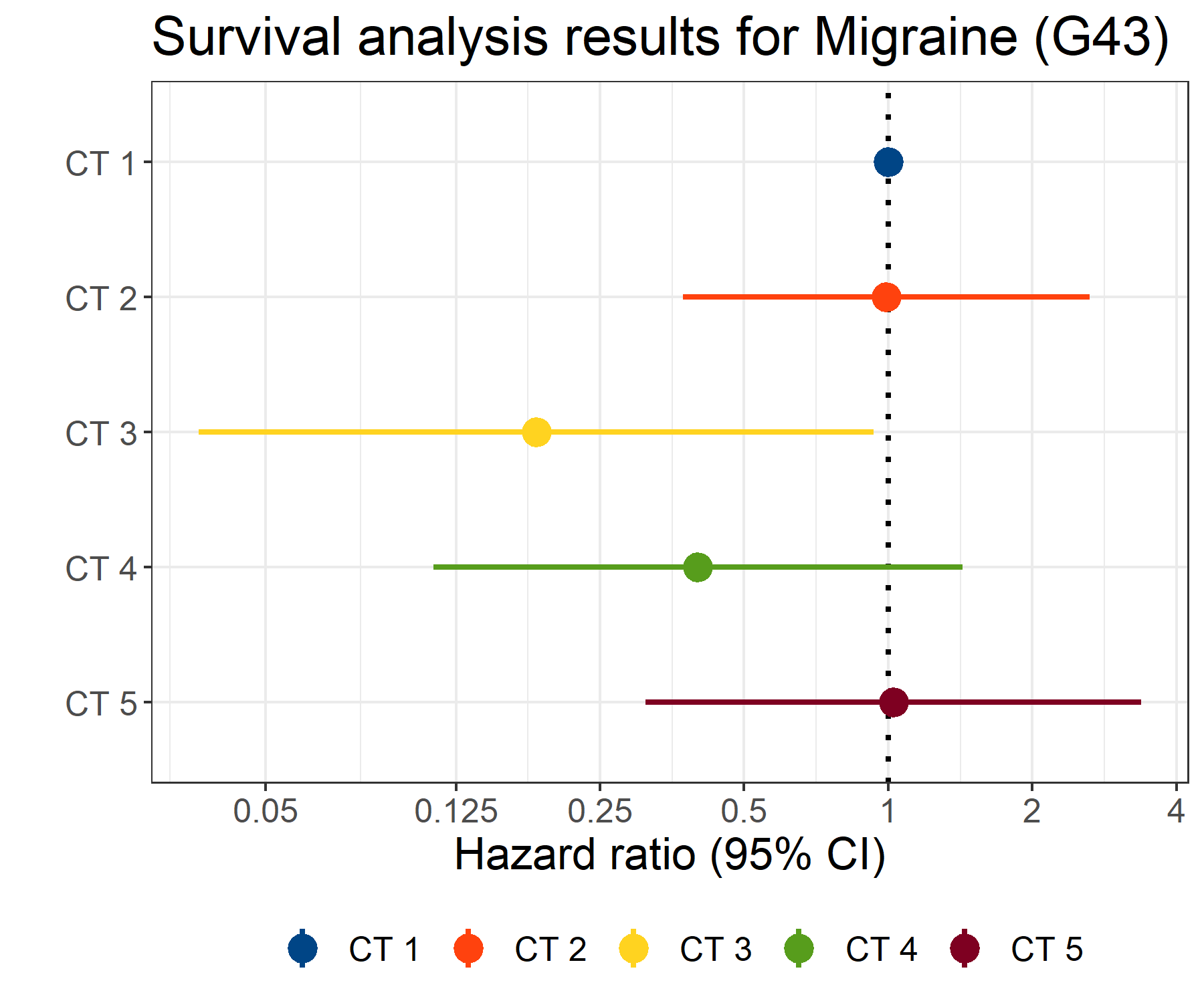


**Supplementary Figure 6.** Survival analysis results for Migraine (G43)
